# Magnetic Resonance Imaging of Enthesitis in Spondyloarthritis, Including Psoriatic Arthritis—Status and Recent Advances

**DOI:** 10.3389/fmed.2020.00296

**Published:** 2020-06-30

**Authors:** Ashish J. Mathew, Mikkel Østergaard

**Affiliations:** ^1^Copenhagen Center for Arthritis Research (COPECARE), Center for Rheumatology and Spine Diseases, Glostrup, Denmark; ^2^Department of Clinical Medicine, Faculty of Health and Medical Sciences, University of Copenhagen, Copenhagen, Denmark; ^3^Clinical Immunology and Rheumatology, Christian Medical College, Vellore, India; ^4^Centre for Prognosis Studies in the Rheumatic Diseases, Krembil Research Institute, University Health Network, University of Toronto, Toronto, ON, Canada

**Keywords:** magnetic resonance imaging (MRI), enthesitis, spondyloarthritis, psoriatic arthritis, inflammation

## Abstract

Enthesitis, inflammation at the attachment sites of tendons, ligaments, fascia, and joint capsules to bones plays a critical role in the pathogenesis of spondyloarthritis (SpA), including psoriatic arthritis (PsA). Magnetic resonance imaging (MRI) has aided in a better understanding of pathophysiology, early diagnosis, prognostication, therapeutic outcomes, and follow up of enthesitis. The concept of enthesitis as a focal insertional pathology has transformed over the past decade, with the help of MRI, to a more widespread entity involving both bone and surrounding soft tissues. The utility of MRI in the differential diagnosis of suspected enthesitis has recently been explored. With the emergence of the treat-to-target concept, and a domain-based approach in the management of SpA, objective and sensitive monitoring of response to targeted therapy becomes prudent. Properties like high sensitivity, ability to image intra-osseous pathology along with surrounding structures exemplify the utility of MRI technology. Considering the lack of a comprehensive, validated MRI score the Outcome Measures in Rheumatology (OMERACT) MRI in Arthritis Working Group, informed by a systematic literature review, developed the first international, consensus-based MRI-scoring system, combined with MRI definitions of pathologies for enthesitis in patients with spondyloarthritis (SpA) and PsA. An atlas with representative images of each grade of the scoring system was subsequently developed by the group to aid readers interested in using the heel enthesitis MRI scoring system (HEMRIS). The HEMRIS can find utility in clinical trials targeting enthesitis as the primary outcome. MRI also finds value for global assessment of the total burden of enthesitis. The concept of whole-body MRI (WBMRI), enabling visualization of entheses throughout the body using a single image is relatively new. The MRI whole-body score for inflammation in peripheral joints and entheses (MRI-WIPE) is a promising scoring system, which is undergoing further testing in clinical trials and longitudinal cohorts evaluating global measures of inflammation at entheses. This review discusses the role of MRI in diagnosis and monitoring of enthesitis in SpA and PsA, along with recent advances in the field, based on published literature.

## Introduction

The entheses are insertion sites of tendon, ligament, fascia, or joint capsule into bone. Enthesopathy refers to involvement of entheses due to trauma, degeneration, or in pathological conditions including metabolic syndrome, endocrine disorders, and inflammatory arthritis (1). Inflammation at the entheseal sites, enthesitis plays a cardinal role in the pathophysiology of spondyloarthritis (SpA), including psoriatic arthritis (PsA) (2). Initially thought to be just a focal insertional site, enthesis is better known currently as being part of an “enthesis organ,” with intricate immune-pathogenetic relationship with synovium, substantiated by McGonagle and colleagues as the concept of synovio-entheseal complex (3). Biomechanical stress induced micro-injuries in the synovio-entheseal complex lead to a cascade of inflammatory process in the adjoining fibrocartilage, bursae, synovium, and trabecular bone by interleukin (IL)-23 from macrophages, dendritic cells and innate lymphoid cells—type 3 (ILC3) (4).

The prevalence of enthesitis in SpA, including PsA from various studies has been reported to be 13.6–35%, with Achilles tendon, plantar fascia, and lateral epicondyle insertion being the most common sites (5, 6). Presence of enthesitis has shown to be associated with higher disease activity, disability and incapacity to work, ultimately leading to poor quality of life (6–8). Clinical enthesitis measures including the Leeds enthesitis index (LEI), the Spondyloarthritis Research Consortium of Canada (SPARCC) enthesitis index and the Maastricht Ankylosing Spondylitis Enthesitis Score (MASES) offer poor reliability and sensitivity compared to advanced imaging techniques like ultrasound and magnetic resonance imaging (MRI) (9, 10).

By providing sensitive visualization of the extent of disease, MRI and ultrasound (US) in patients with PsA have shown utility in diagnosis, prognostication, and monitoring of treatment response. (11) The European League Against Rheumatism (EULAR) recommendations for the use of imaging in SpA, built on research-based evidence and expert opinion highlight the role of MRI in diagnosis and monitoring peripheral enthesitis, acknowledging the need for further research to optimize the use of imaging in clinical practice (12).

This review aims at elucidating the role of magnetic resonance imaging (MRI) in better understanding of enthesitis in SpA, including PsA, highlighting the recent advances in this field.

## How Has MRI Contributed to the Understanding of Pathogenesis of Enthesitis in SpA?

Based on their structure and location two types of entheses have been described – fibrous and fibrocartilaginous, with the latter being affected more commonly in SpA (13, 14). MRI, with its potential to visualize both soft tissue and intra-osseous abnormalities has fostered our understanding of the entheseal organ concept by demonstrating extension of enthesitis to adjacent bone and surrounding structures, including fibrocartilage, bursa, fat pad and deeper fascia (15, 16). McGonagle and colleagues described the correlation of HLA-B27 with the degree of MRI bone marrow edema surrounding the entheses in patients with SpA, compared to those with mechanically induced disease (17). The close link between enthesitis and synovitis in swollen peripheral joints, as demonstrated by MRI studies in PsA and SpA has invoked the possibility of enthesitis inciting an inflammatory response within the closely located synovial tissue (18, 19). This augments the hypothesis that enthesitis is a critical lesion in SpA.

The importance of enthesitis in explaining the relationship between the nail and distal interphalangeal joint disease in PsA was studied by Tan et al. using high-resolution MRI and histology, comparing patients with osteoarthritis (OA) and PsA. The MRI inflammation observed over the entire nail bed region was shown to be anatomically associated with an enthesitis organ apparatus, providing a novel explanation for distal interphalangeal joint (DIP) arthritis in PsA patients with nail involvement (20). Yet another study from the same group applying high resolution MRI to explore flexor tenosynovitis in PsA patients with dactylitis observed microscopic enthesitis in miniature pulleys around the flexor tendon, explaining the tenosynovitis, and also the concept of enthesitis in PsA (21). In a recent study Abrar et al. compared high resolution MRI of hands using a 3 T scanner and dedicated 16-channel hand coil in patients with PsA, rheumatoid arthritis (RA) and healthy controls. Compared to the other groups, PsA patients had significantly thicker A1 and A2 flexor tendon pulleys. This study corroborates the role of enthesitis in the pathogenesis of SpA (22).

## Utility of MRI in the Diagnosis of Enthesitis in SpA

**Figure 1:**
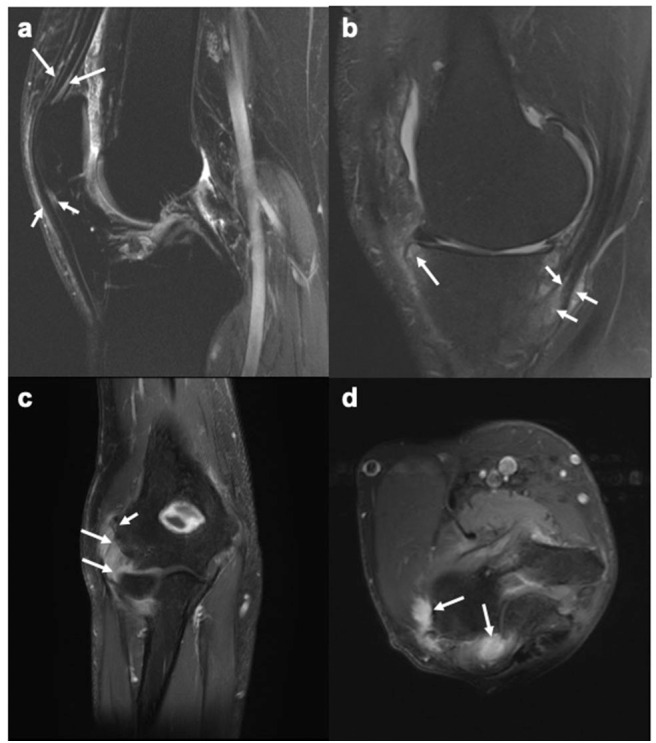
MR images of the knee and elbow depicting enthesitis. **(a)** Sagittal STIR image of the knee showing soft tissue high signal intensity (intra- and peritendinous) at the insertions of the quadriceps tendon (long arrows) and the patellar ligament (short arrows) at the patella, suggesting enthesitis. **(b)** Sagittal T2-weighted fat suppressed image of the knee showing high signal intensity (intra- and peritendinous) in the soft tissues of the pes anserine (short arrows), indicating pes anserine enthesitis, as well as bone marrow edema (long arrows) close to the insertion of the medial patellar retinaculum at medial tibial plateau. **(c,d)** Coronal (c) and axial (d) STIR images of the elbow showing bone marrow edema (mild, short arrow) and soft tissue high signal intensity (long arrows) at the common extensor tendon insertion at the lateral epicondyle, indicating enthesitis. Images courtesy of Professor Iris Eshed, Sackler School of Medicine, Tel Aviv University, Tel Aviv, Israel.

Given the avascular nature of the entheses at bony attachment sites and low density of vessels in the surrounding ligaments and tendons, diagnosis of enthesitis with imaging can be demanding (14). MRI has the unique advantage of identifying peri-entheseal inflammation with adjacent bone marrow edema, potentially facilitating early diagnosis in SpA (23) (Figures 1a–d). Fat-suppressed MRI with or without gadolinium enhancement is the most sensitive method of visualizing active enthesitis (24, 25). The European Society of Musculoskeletal Imaging (ESSR) arthritis subcommittee for the use of MRI has suggested specific sequences based on the area to be examined for inflammatory changes (26). The OMERACT MRI in enthesitis initiative proposes T1weighted post gadolinium sequence for entheseal soft tissue inflammation, STIR/T2weighted fat suppressed sequence for entheseal osteitis, and T1 weighted pre-gadolinium sequence for entheseal structural changes (27). MRI is useful in diagnosing enthesitis in the appendicular and axial skeleton. Bone marrow edema (BME) in PsA is often located close to the entheses, as compared to capsular attachments and subchondral areas in RA and OA, respectively (28).

An MRI and power doppler ultrasound (PDUS) study at the heel region compared SpA patients with current heel pain and those with no pain or past history of heel pain. MRI lesions considered to depict early injury included Achilles tendon (tendonitis) or aponeurosis hypersignal, peri-tendon or peri-aponeurosis hypersignal, retrocalcaneal bursitis, inferior or posterior BME, and thickening of the tendon, and those depicting chronic injury included enthesophytes and bone erosions. MRI pathologies of enthesitis were noted in 81% of SpA patients with current heel pain, compared to 56% of SpA patients without heel pain or with history of heel pain. Intra- or peri-aponeurosis MRI signal abnormalities were the most useful features, while only BME in calcaneum was specific to distinguish patients with SpA from controls (29). A similar study including SpA patients with heel or ankle pain compared high-field and low-field MRI to evaluate the hindfoot. Retrocalcaneal bursitis and plantar fasciitis were the commonest lesions in this study, which inferred an acceptable diagnostic quality for both the units (30). Enthesitis of the rotator cuff, with intense acromial BME at the deltoid origin is described as a highly specific feature of ankylosing spondylitis (AS) (31). According to a recent study erosive changes at the heel entheses seem to be more frequent in peripheral SpA patients compared to non-SpA individuals with painful heels or knees (32).

High resolution MRI with specialized “microscopy coils” have been used for detecting enthesitis at insertions of flexor and extensor tendons to the phalanges. Tan et al. investigated the microanatomic basis for localization of hand disease at the DIP joints in patients with PsA and OA using a high-resolution MRI. More severe changes at the DIP joint entheseal insertions, and marked extracapsular enhancement were noted in patients with PsA as compared to those with OA (33). Another MRI study comparing small joints of hands in RA and SpA patients demonstrated enthesitis and extracapsular changes adjacent to synovial joints more commonly in the latter (34).

**Figure 2:**
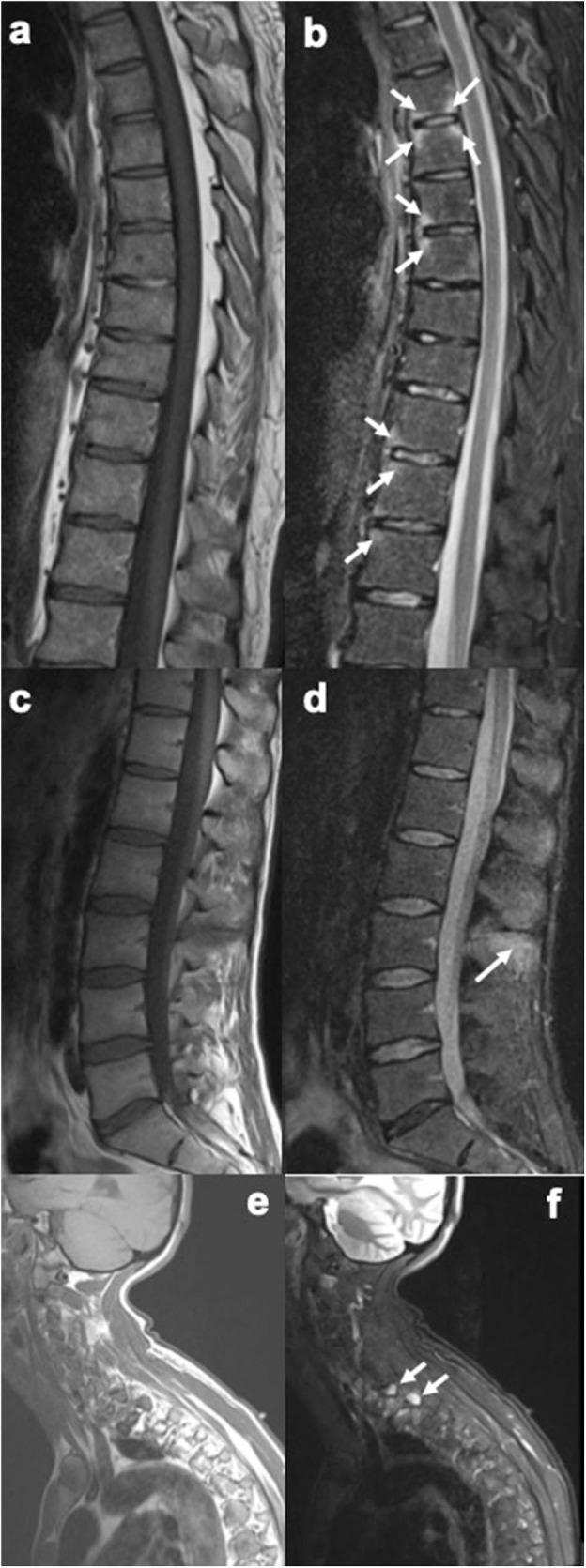
Sagittal MR images of the spine, showing enthesitis at different locations (T1-weighted images on the left, short tau inversion recovery (STIR) images on the right). **(a,b)** Several anterior and posterior corner inflammatory lesions (arrows) are seen in the thoracic spine, representing enthesitis at the insertion of the anterior and posterior longitudinal ligaments. **(c,d)** Bone marrow edema is seen at several spinous processes, particularly at the L3 spinous process (arrow), representing enthesitis. **(e,f)** Bone marrow edema is seen in two upper thoracic transverse processes (arrows), representing enthesitis. Images are from University of Copenhagen, Denmark.

MRI is the imaging method of choice for diagnosing axial enthesitis. The revised definition of MRI enthesitis in sacroiliac joints of patients with SpA excludes the inter-osseous soft tissues in the ligamentary portion of the SI joint (35). Pelvic enthesitis on MRI of sacroiliac joints is highly specific for the diagnosis of SpA, and the specificity increases with the number of sites with enthesitis. Enthesitis at the iliac crest and retroarticular ligaments have high positive predictive value for diagnosis of SpA (36). Spinal enthesitis may be seen on spine MRIs as increased signal intensity over inter-spinal ligaments extending between the transverse or spinous processes, supra-spinal ligaments and osteitis of adjacent bone marrow in the spinous process on short tau inversion recovery (STIR) images, T2-weighted fat-suppressed images and contrast enhanced T1-weighted fat-suppressed images (37). The vertebral corners (or edges) are often inflamed in axial SpA and PsA, and this finding represents enthesitis at the insertions of the anterior and posterior longitudinal ligaments (38) (Figures 2a–f).

Conventional MRI methods generally allow assessment of only one or few selected areas of the human body. Enthesitis in SpA, especially PsA can be widespread, and capturing the extent of disease can be challenging using conventional MRIs (39). Whole-body MRI (WBMRI), with recent technical advancements allows visualization of the entire body in one imaging session. WBMRI may, in the future clinical practice aid in diagnosis of early forms of SpA, enabling evaluation of both axial and peripheral entheses and joints. (40–42) Readability and reproducibility of WBMRI were high in spine and SI joints, but lower in the peripheral joints in earlier studies (43). However, a more recent study has found good reliability in peripheral entheses too (41). Poggenborg et al. investigated the ability of WBMRI to assess axial and peripheral enthesitis in patients with PsA and axial SpA, and observed moderate agreement between clinical examination and WBMRI. The most frequent sites of enthesitis included greater femoral trochanter, supraspinatus and Achilles tendon insertions (10). Weckbach et al. reported enthesitis in 68% of the hip regions among 30 patients with PsA using WBMRI (44). Althoff et al. compared MRI findings in patients with radiographic and non-radiographic axial SpA (nr-axSpA) using WBMRI. Enthesitis, mostly multilocular was significantly more in the SpA group as compared to the nr-axSpA group (45). Weber et al., assessed inflammation at the anterior chest wall using WBMRI in 122 patients, and reported inflammation in 49.5 and 25.9% of patients with SpA and nr-axSpA, respectively as opposed to 26% by clinical assessment using the Maastricht Ankylosing Spondylitis Enthesitis Score (MASES) (46). Though fairly sensitive in its diagnostic capacity, further studies are warranted to demonstrate the ability of MRI in distinguishing inflammatory enthesitis from other forms.

## Role of MRI in Monitoring Inflammatory and Structural Enthesitis in SpA And PsA

Monitoring of disease progression and treatment response is dependent on the responsiveness of the applied measure. MRI has found utility in following up patients with SpA and PsA on treatment. Marzo-Ortega et al., determining the efficacy of etanercept on axial and peripheral entheseal lesions in patients with SpA using MRIs noted improvement or regression in 86% of MRI detected entheseal lesions between baseline and 6 months (47). A similar study by the same group ascertained the efficacy of anakinra on spinal enthesitis in patients with active ankylosing spondylitis (AS) using MRI. There was complete regression or improvement in 23 of the 38 regions of enthesitis (61%) determined by MRI at baseline following 3 months of treatment (48).

Karpitschka et al. demonstrated significant reduction in enthesitis using WBMRI with gadolinium enhancement at week 52 in patients with active AS being treated with etanercept. MRI enthesitis lesions showed reduction during therapy by 94% at week 52 (49). In an investigator initiated randomized controlled trial of adalimumab in patients with axSpA, Krabbe et al. demonstrated the resolution of inflammation at multiple entheseal sites. A higher frequency of clinical resolution was observed in the joints which were tender with MRI inflammation compared to those which were tender without MRI inflammation. This supports the utility of MRI in differentiating inflammatory from non-inflammatory causes of tenderness in patients with SpA (50). Another randomized, double-blind, placebo-controlled trial on adalimumab investigated the responsiveness of WBMRI in axial and peripheral joints and entheses in patients with axSpA. The authors could demonstrate significant reductions in the WBMRI enthesis inflammation index after 6 weeks of adalimumab therapy. The WBMRI total inflammation index, covering both axial and peripheral joints and entheses, could also distinguish treatment from placebo groups (51). Further development and validation of WBMRI inflammation index may be efficacious in assessing responsiveness to treatment of the global entheseal inflammatory burden in future clinical trials.

## Evidence on MRI Enthesitis in Prognostication of SpA And PsA

Existence of a pre-clinical phase in PsA characterized by nonspecific arthralgias, stiffness and fatigue has been established in a prospective cohort study (52). Ultrasound studies have noted baseline sonographic evidence of enthesitis being associated with future development of clinical PsA (53, 54). MRI may aid in detection of the pre-clinical phase of PsA. However, there is scarcity of MRI studies on this topic. Whether pre-clinical MRI enthesitis can predict subsequent development of PsA remains to be established. Enthesitis presumably precedes synovitis in SpA and PsA (3, 55). Peri-entheseal BME at the tibial plateau and bony attachments of patellar tendon and posterior cruciate ligament, detected by McGonagle et al. in SpA and PsA patients with knee swelling of recent onset, indicates subclinical enthesitis near the swollen joint and suggests enthesitis as the primary lesion (18). Emad et al. studied the entheseal changes at knee joints in patients with psoriasis and SpA, including axSpA, inflammatory bowel disease (IBD) and PsA. Subclinical enthesitis was noted in patients with psoriasis and IBD. The authors concluded that enthesitis at the knee joint may be an early and pathognomonic MRI finding in patients with SpA (56). Erdem et al. determined MRI changes of foot in psoriasis patients with no clinical arthritis and healthy controls. Achilles tendonitis and retrocalcaneal bursitis were observed in more than half of the psoriasis patients, with none in the healthy control group having similar findings (57).

These studies, although with significant limitations did provide insight into the possibility of MRI enthesitis being the initial subclinical pathology in SpA. In a recent study Simon et al., scanning psoriasis patients without clinical arthritis from a longitudinal cohort have established the role of structural enthesitis at the 2nd and 3rd metacarpophalangeal joints of the dominant hand in prediction of PsA (58). Another study evaluating MRI inflammation in the hand joints of patients with psoriasis without PsA and healthy controls noted a 55.5% likelihood of developing PsA in psoriasis patients with arthralgia and evidence of MRI synovitis. This observation should, nevertheless be interpreted in the background of a 29.6% conversion rate to PsA in this cohort within the short follow up period of 1 year. Moreover, osteitis, periarticular inflammation and tenosynovitis were comparable in psoriasis patients and controls (59).

## How Have Clinical Trials Applied MRI for Assessing Enthesitis?

With the advent of treat-to-target strategies in SpA and domain specific treatment approach in PsA, a subset of future clinical trials with novel molecules are expected to focus on enthesitis as their primary outcome (60–62). Inclusion of enthesitis as a core domain by the outcome measures in rheumatology (OMERACT) PsA group warrants its assessment in all clinical trials and observational studies (63). MRI, despite being an objective and sensitive adjunct to clinical examination for monitoring response of enthesitis to therapy, has not been employed in clinical trials distinctly. One of the early placebo-controlled, randomized clinical trials of etanercept to determine its efficacy in patients with refractory heel enthesitis used MRI as an adjunct to clinical examination. No statistically significant differences were noted between the placebo and etanercept groups among the 19 patients who presented with positive MRI heel enthesitis, defined by BME at calcaneus insertions of Achilles tendon and plantar fascia (64). The ACHILLES trial is a randomized, quadruple-blind study (NCT02771210) evaluating the efficacy of secukinumab in resolution of Achilles tendon enthesitis in patients with active PsA and axSpA in which MRI is applied as a secondary outcome measure. The recruitment of this trial has been completed and the results are awaited (65).

Improvement in overall enthesitis in the body, as assessed by WBMRI, have been studied in few clinical trials. A randomized clinical trial compared etanercept and sulphasalazine on active bony inflammation in patients with early axSpA using WBMRI. Reduction of peripheral enthesitis on MRI was a secondary endpoint. The authors demonstrated a 58% reduction in MRI enthesitis in patients on etanercept at week 48, as compared to no reduction in the comparator arm (66). Another randomized, placebo-controlled trial to investigate efficacy of adalimumab on WBMRI indices of inflammation at entheses in patients with axSpA demonstrated significant reduction of both BME and soft tissue indices in the treatment group compared to placebo at week 6 (51).

For MRI assessment of enthesitis in clinical trials, it is recommended to apply validated assessment methods, such as the OMERACT MRI scoring systems (27, 41). More studies are needed to validate and optimize the existing MRI outcome measures. Different aspects of validity, including criterion validity (comparison with gold standard reference, such as histopathology) and discriminant validity (reproducibility and sensitivity to change) should preferably be investigated.

## Utility of MRI in Enthesitis in Juvenile Arthritis

Active enthesitis and arthritis in patients with enthesitis related arthritis (ERA) at baseline has been reported to predict sacroiliitis at follow up (67). Enthesitis detected by MRI of the pelvis has been described as a specific finding in juvenile spondyloarthritis (68). Herregods et al. determined the diagnostic value of enthesitis on pelvic MRIs in patients with ERA, and noted high correlation between pelvic enthesitis and sacroiliitis (69). WBMRI is increasingly being used in the pediatric population to determine the overall inflammatory and structural burden of synovitis and enthesitis (70). Enthesitis has been included in the inflammatory MRI components of the recently developed OMERACT juvenile idiopathic arthritis MRI score (71).

## Limitations of Imaging Enthesitis in SpA and PsA Using MRI

Notwithstanding all these benefits, MRI has certain limitations which curtails its application in routine clinical settings. Practical impediments for clinical practice like cost, referrals to specialist facilities, and some contraindications, such as claustrophobia, pacemakers, or certain metal implants, cannot be overlooked. The avascular nature and limited water accumulation in structures that make up the entheses contribute to technical difficulties, with MR signals often being low (72). A major limitation with MRI enthesitis until recently was the lack of a comprehensive, generally accepted, validated scoring system with proper definition of pathologies to be scored, which can be applied uniformly in all clinical trials and longitudinal observational studies. Most scoring methods and lesions adapted in studies of MRI enthesitis display poor content and construct validity, and lack responsiveness (73).

Limitations with WBMRI include the examination time, low resolution of images and attainable spatial resolution compared to conventional MRI (74, 75). The total scan time for WBMRI, including peripheral and axial joints, and entheses is generally around 60 min (41, 43). Patients with active arthritis may find it challenging to remain stationary in the same position for long periods of time, which may result in motion artifacts. Image resolution with current acquisition techniques could be compromised, especially in the distal small joints. With advances in technology these limitations could be addressed to a great extent. Experience of the reader also plays a pivotal role with WBMRI. Reliability among experienced readers has been shown to be good, while poorer among less experienced ones (41).

For use in clinical trials, however, MRI has the major advantage of allowing fully standardized image acquisition across all study sites, storage of the entire examination for later review and centralized reading. This makes MRI the ideal method for objective assessment of entheseal inflammation in future clinical trials.

## Recent Advances in the Field of MRI Enthesitis in SpA

Expected advancements countering the technical shortcomings of MRI in imaging entheses have recently been reported. Diffusion-weighted imaging (DWI), known to have a high signal to noise ratio has been analyzed as an encouraging alternative to STIR and T2 weighted fat suppressed sequences for sacroiliac joint assessment (76–78). Lecouvet et al. compared the diagnostic accuracy of DWI and STIR sequences in WBMRI of SpA patients. DWI was found to offer higher sensitivity for detection of inflammatory lesions compared to STIR sequences, and to differentiate inflammatory from degenerative changes (78). Ultrashort echo time (UTE) sequences have been explored for better visualization of entheses. Chen et al. demonstrated higher resolution of enthesis at the Achilles tendon in healthy volunteers and patients with PsA using three-dimensional UTE-cones sequences, compared to gradient recalled echo (GRE) and fast spine echo (FSE) sequences. The authors stressed the utility of this sequence in morphological and quantitative evaluation of enthesitis in PsA patients (79). The same group recently explored the MRI morphology of Achilles tendons and entheses using high resolution MRI UTE sequences, and described its utility as biomarkers of biomechanical degradation of entheses in SpA (80).

**Figure 3:**
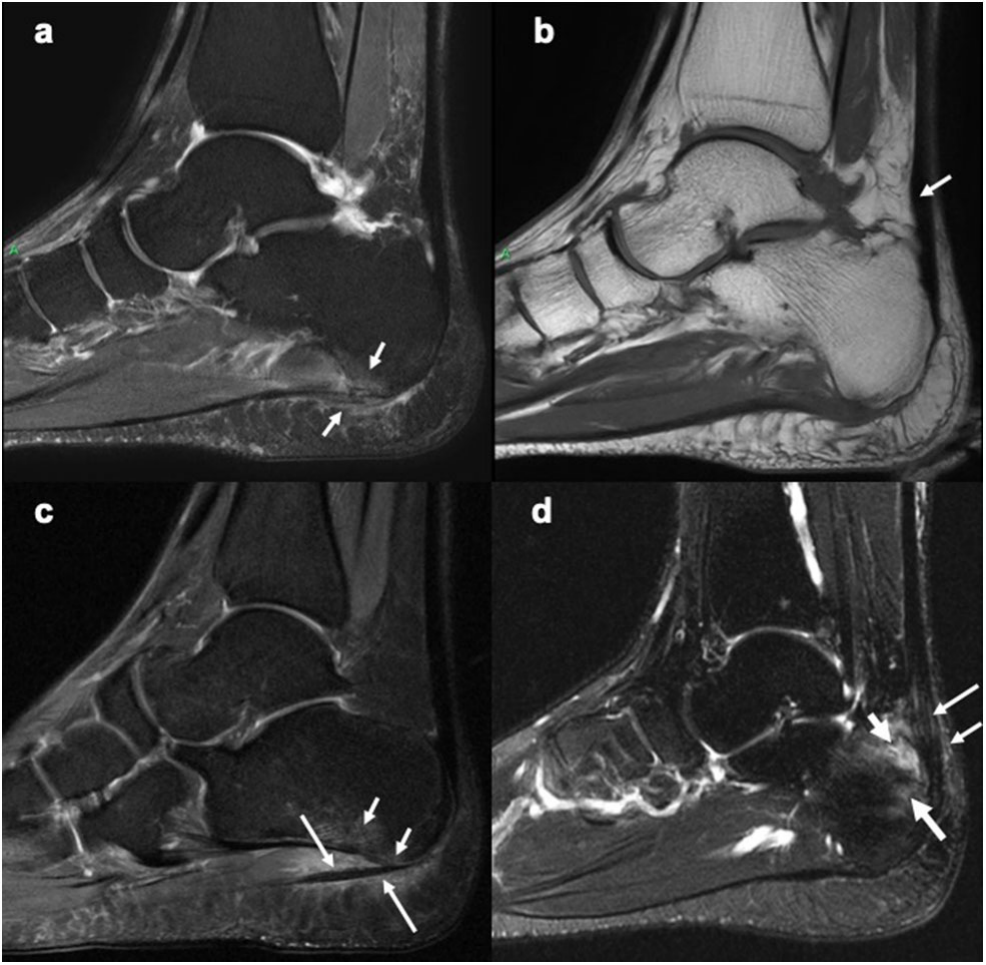
Sagittal MR images of the heel region depicting enthesitis at Achilles tendon and plantar fascia attachments. **(a)** STIR image showing bone marrow edema at the plantar fascia insertion to the calcaneum, intrafascia high signal intensity and perifascia high signal intensity. **(b)** Corresponding T1-weighted image showing probable mild thickening of a part of the Achilles tendon (arrow). **(c)** STIR image showing bone marrow edema (short arrows) close to the plantar fascia insertion to calcaneum and severe perifascia high signal intensity (long arrows). **(d)** STIR image showing bone marrow edema (long thick arrow) at the Achilles tendon insertion to calcaneum, intratendonous (long thin arrow) and, peritendonous (short thin arrow) high signal intensity, as well as retrocalcaneal bursitis (short thick arrow). **(a,b)** Courtesy of Professor Iris Eshed, Sackler School of Medicine, Tel Aviv University, Tel Aviv, Israel. **(c,d)** have been taken from the OMERACT MRI heel enthesitis exercises (27, 80).

The OMERACT MRI in arthritis Working Group, informed by a systematic review has developed consensus-based definitions and reader rules for enthesitis in patients with SpA and PsA. Through a series of multi-reader scoring exercises focusing on the heel region using an intuitive web-based image platform and data entry the group developed the OMERACT heel enthesitis in MRI scoring system (HEMRIS). HEMRIS exhibited good reliability and responsiveness among trained readers (27). This was followed by an atlas of the OMERACT HEMRIS, with detailed definitions and reader rules (Box 1), which could be used as a guide while scoring Achilles tendon and plantar fascia enthesitis (Figures 3a–d) in future clinical trials and longitudinal studies using MRI (81). Applying a similar methodology the group also developed a WBMRI scoring system (MRI-WIPE) for peripheral arthritis and enthesitis, which depicted good reliability among the experienced readers (41, 42).

Box 1 **OMERACT HEMRIS recommendations for MRI acquisition, definitions and scoring of inflammatory and structural pathologies at the entheses (Adapted from Box 1, Mathew et al. (81)).**

**A. Core set of basic MRI sequences and imaging planes**

MRI studies that intend to assess inflammatory and structural changes at entheses should include at least the following sequences:

- Short tau inversion recovery (STIR)/T2-weighted fat suppressed (T2wFS) images or, alternatively, gadolinium-enhanced T1-weighted fat suppressed images

- T1-weighted images without contrast injection (not mandatory if only inflammation is being assessed)

Suggested imaging planes for the heel region:

- Achilles tendon—Sagittal and preferably also axial

- Plantar fascia—Sagittal and preferably also coronal

**B. Definitions and grades of inflammatory and structural pathologies at the Achilles tendon insertion and the plantar fascia insertion to the calcaneum** (Adapted from Box 1, Mathew et al. (81))

***1. Intratendon/intrafascia hypersignal (STIR/T2FS)***

**Definition**

Signal characteristics consistent with increased water content/inflammation within the tendon/fascia, close to its insertion^¥^.

**Grades**

0: No intratendon/intrafascia hypersignal. *

1: Minimal intratendon/intrafascia hypersignal spots * (≤ 25% of the tendon volume).

2: Moderate intratendon/intrafascia hypersignal * (>25% and ≤ 50% of the tendon volume).

3: Severe intratendon/intrafascia hypersignal * (>50% of the tendon volume).

^¥^ For Achilles tendon: From the tendon insertion up to 2 cm proximal to the posterosuperior corner of calcaneum on all the available images. For Plantar fascia: From the fascia insertion up to 2 cm proximal to the anterior margin of the plantar tuberosity on all the available images.

***2. Peritendon/perifascia hypersignal (STIR/T2FS)***

**Definition**

Signal characteristics consistent with increased water content/inflammation in the soft tissues surrounding the tendon or fascia, close to its insertion.

**Grades**

0: No hypersignal. *

1: Minimal^*†*^ (or mild) focal hypersignal. *

2: Moderate^*†*^ hypersignal. *

3: Severe^*†*^ hypersignal. *

^*†*^*By comparison with reference images (see Mathew et al*. *(**81**))*

* For Achilles tendon: From tendon insertion up to 2 cm proximal to the posterosuperior corner of calcaneum. For Plantar fascia: From fascia insertion up to 2 cm proximal to the anterior margin of the plantar tuberosity on all the available images.

***3. Achilles tendon/plantar fascia calcaneal bone marrow edema***

**Definition**

Bone marrow edema (BME) should be assessed in the bone from the entheseal insertion to a depth of 1 cm on all available images.

Grades:

The scale is 0-3, based on the proportion of bone with edema, compared to the “assessed bone volume”, judged on all available images:

0: no edema.

1: 1–33% of the bone is edematous (i.e. BME occupying 1–33% of the assessed bone volume).

2: 34–66% of the bone is edematous.

3: 67–100% of the bone is edematous.

If the lesion is judged borderline, i.e., 1 vs. 2 or 2 vs. 3, lesion intensity may be considered. For example, if a lesion is borderline between 1 (mild) and 2 (moderate), it may be scored 1 (mild) if not judged intense. Similarly, if a lesion is borderline between 2 (moderate), and 3 (severe), it may be scored 3 (severe) if judged intense.

***4. Retrocalcaneal bursitis (only relevant at Achilles tendon insertion)***

**Definition**

Signal characteristics consistent with increased water content/inflammation in an above-normal sized bursa.

**Grades**

0: No hypersignal or maximal diameter of hyper-signal in the shorter of two perpendicular dimensions to be <0.25 cm.

1: Maximal diameter of hypersignal in the shorter of two perpendicular dimensions to be ≥0.25 cm to <0.5 cm.

2: Maximal diameter of hypersignal in the shorter of two perpendicular dimensions to be 0.5 cm to <1.0 cm.

3: Maximal diameter of hypersignal in the shorter of two perpendicular dimensions to be ≥1.0 cm.

***5. Tendon/fascia thickening***

**Definition**

Abnormal thickening of the tendon/fascia close to its insertion. *

**Grades**

0: None.

1: Mild.^*†*^ *

2: Moderate.^*†*^ *

3: Severe.^*†*^ *

^*†*^*By comparison with reference images (see Mathew et al*. *(**81**))*

* For Achilles tendon: Maximally 2 cm proximal from the postero-superior corner of calcaneum. For plantar fascia: Maximally 2 cm proximal to the anterior margin of the plantar tuberosity.

***6. Achilles tendon/plantar fascia calcaneal enthesophyte***

**Definition**

Abnormal bone formation at the insertion of tendon/fascia into the bone

**Grades**

0: None.

1: Small.^*†*^

2: Medium-sized.^*†*^

3: Large.^*†*^

^*†*^*By comparison with reference images (see Mathew et al*. *(**81**))*

***7. Achilles tendon/plantar fascia calcaneal bone erosion***

**Definition**

A sharply marginated bone lesion, with typical signal characteristics and a visible cortical break, located close to the tendon/fascia insertion.

***Grades***

0: None.

1: Small.

2: Medium-sized.

3: Large.

*By comparison with reference images (see Mathew et al*. *(**81**))*

## Future Perspectives

### Technical Advancements

New and potentially better MRI sequences have been described in SpA, but they need validation in longitudinal studies and clinical trials, applying novel and established MRI enthesitis measures in the same studies, to document validity and additional value as outcome measures. Also, their utility in assessing axial and peripheral entheses, and differentiating inflammatory from degenerative and other causes of enthesitis needs to be appraised in larger number of patients. The UTE sequence in high resolution MRI may help in better understanding of pathogenesis of different forms of enthesitis. There seems to be a window of opportunity in patients with SpA and PsA wherein early diagnosis and prompt initiation of therapy will be key in curtailing long-term damage (82). The utility of pre-clinical enthesitis visualized by MRI in prognostication of future PsA or SpA needs validation.

### Refinement and Further Validation of MRI Scoring Systems

The recently developed MRI enthesitis indices should be further tested in clinical trials. Refinements may further improve their utility. Development of detailed MRI scoring systems for other regions than the heel may be relevant, even though it seems likely that clinical trials aiming to document the effect of a new drug on enthesitis will choose either a detailed evaluation of the most common region, like the heel, or an overall measure of enthesitis in the entire body, by WBMRI. Image resolution in WBMRI needs further enhancement for better visualization of peripheral entheses. The OMERACT MRI in arthritis Working Group is currently endeavoring on a modular approach in WBMRI to assess the overall inflammation burden at individual sites, thus further validating the WIPE-MRI scoring system. Based on the definitions laid out by the Working Group scoring systems for other regions need to be developed.

### Role of MRI in Disease Prediction and Interception

In the transition phase toward the development of PsA, a subclinical phase with soluble biomarkers and imaging findings but no clinical sign is well recognized (83). Longitudinal studies with high-resolution MRI of hand or foot at baseline in psoriasis patients at risk of developing PsA are warranted to further validate the role of imaging in predicting PsA. For instance, WBMRI could be utilized in quantification of the global inflammatory burden of enthesitis in psoriasis patients at risk of developing PsA, and it could be investigated if this overall enthesitis burden is closely related with future development of PsA in the “at risk” patients.

Effect of treatment strategies to impede the development of PsA in psoriasis patients at risk may also be estimated. In the Interception in very early PsA (IVEPSA) study, MRI was used to assess inflammatory and structural changes in the joints at baseline and 24 weeks following secukinumab therapy (84). Most of the intervention studies with this objective focus on imaging synovitis in the hand joints. Nevertheless, technical advancements and validated scoring systems like the HEMRIS and WIPE-MRI can pave the way for harnessing MRI enthesitis as an outcome in future clinical trials targeting disease interception.

## Conclusion

MRI has aided in understanding the pathogenesis, assessment of inflammatory and structural pathologies, monitoring, and prognostication of enthesitis in patients with SpA, including PsA. There is robust evidence for MRI as an adjunct to clinical examination in the assessment and follow up of enthesitis. Having a sound knowledge of its strengths and weaknesses, as compared to other imaging modalities, will facilitate optimal application of MRI in clinical trials, longitudinal studies and clinical practice.

## Author's Note

All the figures in this manuscript are unpublished, and original. These figures are based on the “MRI definitions of key entheseal pathologies” as described by the OMERACT MRI in arthritis Working Group in Table 1 of Mathew et al. (27).

## Author Contributions

AM prepared the first draft of the manuscript, and MØ critically reviewed and modified the manuscript. All authors contributed to the article and approved the submitted version.

## Conflict of Interest

The authors declare that the research was conducted in the absence of any commercial or financial relationships that could be construed as a potential conflict of interest.
